# Gold-coated plant virus as computed tomography imaging contrast agent

**DOI:** 10.3762/bjnano.10.195

**Published:** 2019-10-07

**Authors:** Alaa A A Aljabali, Mazhar S Al Zoubi, Khalid M Al-Batanyeh, Ali Al-Radaideh, Mohammad A Obeid, Abeer Al Sharabi, Walhan Alshaer, Bayan AbuFares, Tasnim Al-Zanati, Murtaza M Tambuwala, Naveed Akbar, David J Evans

**Affiliations:** 1 Yarmouk University - Faculty of Pharmacy, Department of Pharmaceutical Sciences, Irbid, Jordan; 2 Yarmouk University - Faculty of Medicine, Department of Basic Medical Sciences, Irbid, Jordan; 3 Yarmouk University - Faculty of Science, Department of Biological Sciences, Irbid, Jordan; 4 Hashemite University - Department of Medical Imaging, Zarqa, Jordan; 5 National Agricultural Research Center, Amman, Jordan; 6 University of Jordan - Cell Therapy Center, Amman, Jordan; 7 SAAD Centre for Pharmacy and Diabetes, School of Pharmacy and Pharmaceutical Science Ulster University, Coleraine, UK; 8 University of Oxford - Cardiovascular Medicine, John Radcliffe Hospital, Oxford, UK; 9 John Innes Centre, Norwich Research Park, Norwich, UK

**Keywords:** biomedical imaging, computed tomography (CT), gold, nanotechnology, viruses, targeting

## Abstract

Chemical modification of the surface of viruses, both the interior and the exterior, imparts new functionalities, that have potential applications in nanomedicine. In this study, we developed novel virus-based nanomaterials as a contrast agent for computed tomography (CT) imaging in vitro. The gold-coated cowpea mosaic virus (Au-CPMV) particles were generated by the electrostatic adsorption of positively charged electrolyte on the virus capsid with the subsequent incubation and reduction of anionic gold complexes. Au-CPMV particles as a CT contrast agent offer a fast scan time (less than 2 min), low cost, and biocompatibility and allow for high-resolution imaging with ca. 150 Hounsfield units (HU). The Au-CPMV surface was further modified allowing for the incorporation of targeting molecules of specific cell types.

## Introduction

Numerous types of nanomaterials are currently under investigation in medicine, including dendrimers, polymeric nanoparticles (NPs), liposomes and protein-based NPs. Each system has advantages and disadvantages in terms of its toxicity, biocompatibility, immunogenicity, distribution and the payload being carried.

Modified protein cages are robust systems that combine imaging capabilities and target selectivity on the same platform. One application is the development of magnetic resonance imaging (MRI) contrast agents. Current contrast agents achieve their effect by increasing the relaxation rates (longitudinal relaxation rate (*R*_1_), transverse relaxation rate (*R*_2_), and pseudo-transverse relaxation rate (*R*_2_*)) of water protons in tissues through the catalysis of alignment of nuclear spins [1], thus manipulating the MR image contrast. This effect is known as paramagnetic relaxation enhancement [2] and is common among contrast agents containing gadolinium [3] and iron oxide nanoparticles [4]. CT is a non-invasive, diagnostic imaging tool that allows for 3-D visual reconstruction and tissue segmentation. It relies on the use of X-rays with wavelengths between 0.01 nm and 10 nm [5]. The CT image is generated from the 360° rotation of the X-ray beam source around the object, with a detector positioned opposite to the radiation source. The obtained attenuation profiles are processed mathematically by algorithms to create a 3-D image reconstructed from the dataset of the scanned object and expressed in Hounsfield units (HU) [6]. X-ray attenuation and the image contrast result from the scattering (differential) of the X-rays in the tissue. Tissue and bones absorb X-rays more strongly than air [7].

NPs hold great potential as molecular imaging tools [8]. In general, NPs carry high contrast agent payloads in comparison to smaller moieties [9]. Semiconductor quantum dots (QDs) are nanosized crystals, a photostable fluorophore with a broad excitation spectrum but narrow emission at wavelengths dependent on the size and chemical composition of the core [10]. NPs, such as QDs and magnetic NPs, generate a contrast signal that is unmatched by smaller chemical counterparts [11]. Although iodine-based contrast agents are the most commonly used CT contrast agents nowadays [12], a variety of materials have been used as CT contrast agents including gold nanoparticles (AuNPs) [13], bromine [14], platinum [15], ytterbium [16], gadolinium [4], and tungsten [15]. Many of the systems are made up of a core that is coated with a polymeric material such as liposomes [17], micelles [13], lipoproteins or polymeric nanoparticles [18]. One of the first examples of such NP-based systems was reported by Caride et al. using brominated phospholipids packaged into liposomes and administered to dogs. Contrast enhancement signals of 40 HU were observed in the liver of imaged animals [14]. Two hours after injection, micelles loaded with 17.7 wt % of iodine at a dose of 170 mg of iodine per kilogram were able to show an attenuation of 150 HU in the heart [19], 57 HU in the liver and 90 HU in the spleen [20].

The development of AuNPs as imaging agents was invigorated after Hainfeld reported the formulation of a 1.9 nm contrast X-ray imaging agent after the injection of rats with 2.7 g gold per kilogram with no observable toxic effects [21]. Cai et al. synthesized AuNPs coated with PEG-2000 with a hydrodynamic radius of 38 nm and a 10 nm core [22] that generated a 100 HU signal in the aorta at a dose of 493 mg of gold per kilogram with a mean circulation half-life of 14.6 h. Von Maltzahan et al. developed PEGylated gold nanorods (13 × 47 nm) as CT contrast agents and for photothermal tumor therapy. The study resulted in tumor elimination and mice survival over 50 days [23]. Further, van Schooneveld et al. reported micelle-based AuNPs by generating 66 nm AuNPs coated with an 11 nm layer of silica and showed that for mice injected with these particles a contrast signal was observed [13]. Popovtzer et al. reported successful CT imaging of squamous cell carcinoma using gold nanorods coated with anti-antigen A9 [24].

The low sensitivity of contrast media represents a major challenge in the targeted CT imaging approach [23]. The minimum detectable signal was defined by Krause [25] to be 30 HU [26], with an attenuation rate of gold being 5.1 HU and with a concentration difference of 5.9 mM between the target and the background noise [27]. The accumulation of such concentration of the contrast agent is very difficult [28]. Therefore, the need to develop different forms on nanoparticles that are densely loaded with CT contrast agents for use in clinical settings will be of great value. The work reported here explores the development of a plant virus-based NP as a CT imaging agent.

In this study, the plant cowpea mosaic virus (CPMV) was coated with a gold shell and the use as a CT contrast agent was evaluated. Although a few publications reported the decoration of the surface of virus capsids with preformed AuNPs in specific patterns [29], to the best of our knowledge, this is the first time that modified plant virus particles have been used for CT imaging in vitro; the generated particles have potential for clinical imaging applications.

## Results and Discussion

### Characterization of Au-CPMV

**Figure 1:**
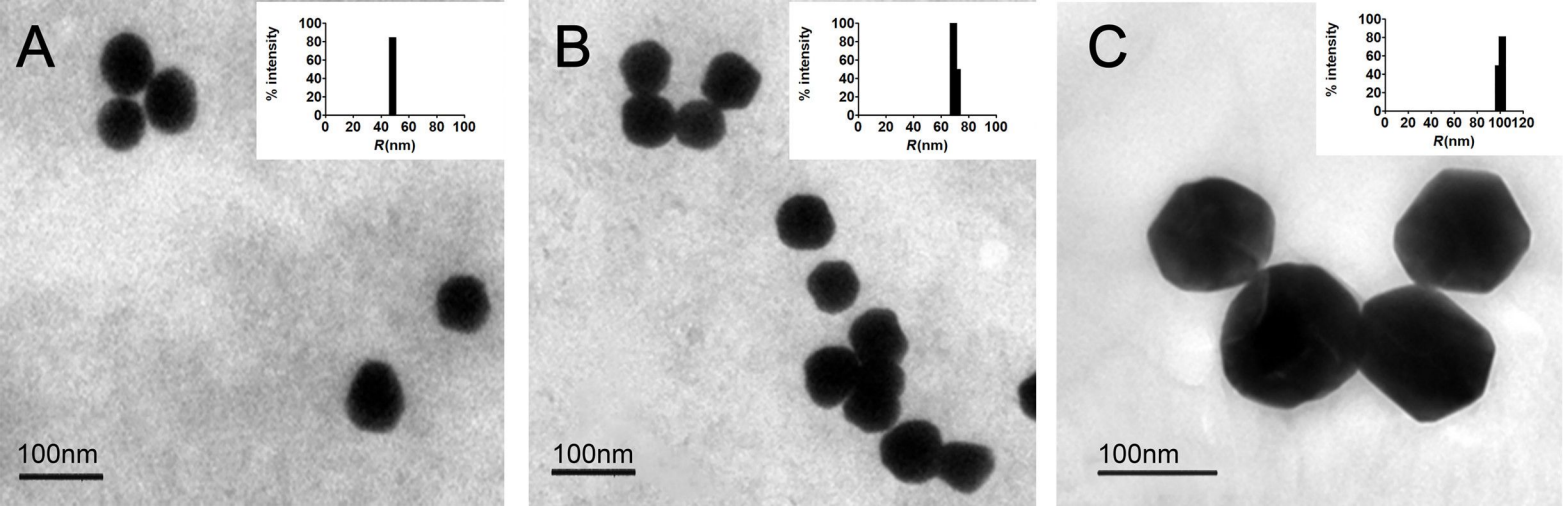
Unstained TEM images of Au-CPMV with the corresponding DLS size distribution histograms (inset). (A) 50 nm Au-CPMV particles; (B) 70 nm Au-CPMV particles; (C) 100 nm Au-CPMV.

A colloidal solution of Au-CPMV was synthesized based on the previously described method [30], by the adsorption of positively charged polymer on the CPMV virus capsid followed by subsequent reduction of gold ions onto the virus capsid. The approach allowed for the control of the size and generated a highly monodisperse distribution with limited coalescence. Au-CPMV assemblies were freely suspended; no aggregation was observed by nanoparticle tracking analysis (NTA) or dynamic light scattering (DLS). The successful coating of CPMV particles with gold was confirmed by TEM (Figure 1). Nearly spherical NPs were observed the average diameters of which are given in Table 1. Au-CPMV particles imaged by TEM appeared smaller in size than in NTA and DLS measurements, because TEM measures the solid cores of the particles. Three different sizes of NPs were generated (50, 70 and 100 nm) by varying the amount of gold hydroxide. The Au-CPMV particles were dark red in color. Characteristic SPR bands of Au-CPMV were observed in the UV–vis spectrum at λ = 535 nm for particles with a diameter of 50 nm, at λ = 552 nm for particles with a diameter of 70 nm and at λ = 572 nm for particles with a diameter of 100 nm (see below in Figure 3C). This confirms the formation of spherical particles for all three sizes. The surface plasmon resonance depends on the shape and the size of the NPs. For instance, ellipsoid shapes with three different axes have three different dipole modes. When the size of the spherical AuNPs increases, their SPR does not red-shift significantly. However, when the spectrum of rod-shaped particles is recorded the SPR shifts dramatically. The central resonance peak around 520–530 nm represents the transverse SPR, which corresponds to the electron oscillation [31] vertically to the long axis and it coincides spectrophotometrically with the SPR spectrum of spherical nanoparticles [32].

The hydrodynamic diameter and the polydispersity of the Au-CPMV suspended colloidal particles were measured by DLS and NTA. Bare unfunctionalized particles showed a hydrodynamic diameter and polydispersity index (PDI) values of the Au-CPMV particles are listed in Table 1. The values are in accordance with the size observed from the TEM images and further confirms the narrow size distribution of the three types of Au-CPMV particles. The particle size measured by DLS is influenced by the substances adsorbed on the NP surface and by the electrical double layer (solvation shell). Therefore, the size measured in DLS instruments appears to be slightly bigger than that measured by TEM [33]. Particles with PDI values below 0.1 are considered highly monodisperse [33]. Furthermore, to confirm the monodispersity of the particles the Cumulants fit correlogram generated by the DLS instruments, that measures the time at which the correlation starts to significantly decay, gave a slope of 85.3° consistent with a monodisperse distribution. The steeper the line (closer to 90°) the more monodisperse the particles are.

**Table 1:** Size, particle count and zeta potential of bare (unfunctionalized) particles, functionalized NPs and CPMV. The concentration of the Au-CPMV particles was 0.5 mg/mL gold for all three particle sizes. Particle count (number of particles per mL) as measured by NTA, *n* = 6 measurements.

particles	TEM (nm)	DLS		NTA (nm)	particle count (× 10^9^/mL)	zeta potential (mV)
		Z-ave (nm)	PDI (nm)			

CPMV	27 ± 2.0	30 ± 1.1	0.05 ± 0.01	28.9 ± 1	3.56	−13

Au-CPMV (50 nm)	44.5 ± 4.2	50.2 ± 3.2	0.04 ± 0.01	51 ± 2	2.32	−45.9 ± 3.1
Au-CPMV (70 nm)	63.5 ± 4.0	68.0 ± 2.0	0.13 ± 0.03	71 ± 3	1.87	−48.2 ± 1.8
Au-CPMV (100 nm)	92.0 ± 3.8	96.0 ± 4.1	0.15 ± 0.08	100 ± 5	1.08	−43.7 ± 2.1

^PEG 5000^Au-CPMV	—	50.2 ± 3.2	0.12 ± 0.07	—	—	−30.2 ± 2.1
^VCAM1-PEG5000^Au-CPMV	—	56.1 ± 2.4	0.16 ± 0.02	—	—	−20.5 ± 1.2

The zeta potential cannot be measured directly, rather it is deduced from the electrophoretic mobility of the charged NPs under an applied electric field. The electrophoretic mobility toward the positive or the negative electrode determines the zeta potential values as negative or positive. The zeta potential values for Au-CPMV particles of different suspensions are summarized in Table 1. The zeta potential is consistent, in each case, with the formation of a similar layer deposited on the surface of the Au-CPMV particles [34]. The zeta potential values of unfunctionalized Au-CPMV agrees with previously reported values [35] ranging between −43.7 ± 2.1 mV and −48.2 ± 1.8 mV [36]. The zeta potential of ^VCAM1-PEG5000^Au-CPMV is −20.5 ± 1.2 mV, that of ^PEG 5000^Au-CPMV is −30.2 ± 2.1 mV. One of the advantages of zeta potential measurements is the possibility to classify NP stability based on the surface charge values. NPs with values in the range of ±30 mV are considered highly stable [37]. The high values of zeta potential observed here confirm the electrostatic repulsion between the NPs that increases their stability and extends their shelf life. The zeta potential of the particles measured after 10 months storage at 4 °C gave similar values.

**Figure 2:**
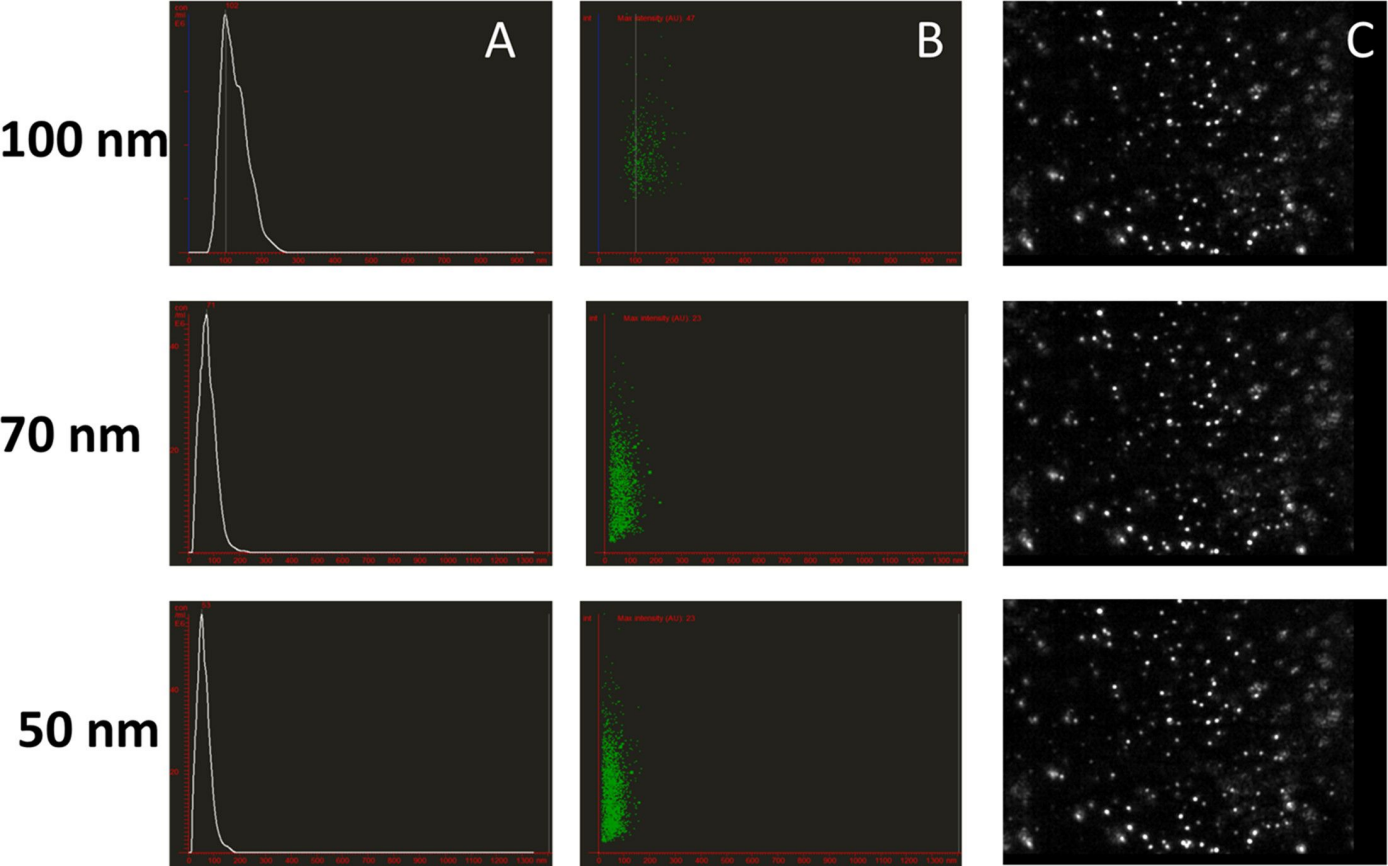
NTA measurement of Au-CPMV at 25 °C recorded from three consecutive runs (60 s) for each sample. (A) particle size (concentration particles/mL as a function of the size); (B) representative NTA scattering distributions of each population (size as a function of intensity) showing compact clustering and indicating highly monodisperse particles with no aggregation, each dot represents a single particle; (C) representative NTA video frame. Data was recorded from six independent experiments.

NTA analysis of the Brownian motion of the Au-CPMV samples on a particle-by-particle basis and the subsequent employment of the Stokes–Einstein equation allows for the derivation of NP size and concentration. Au-CPMV with a concentration of ca. 0.5 mg/mL gold contains roughly 10^9^ to 10^10^ Au-CPMV particles per milliliter as determined by NTA. The solution was diluted to obtain ca. 3500 NPs in the quartz cuvette (300 μL) of the instruments under laminar flow. Of these 3500 NPs ca. 100–200 NPs are illuminated at any given time as determined from still images of the recorded video using ImageJ software. The particle size distribution obtained from NTA analysis (Figure 2A) showed a peaks of 51 ± 2 nm, 71 ± 3 nm and 100 ± 5 nm, respectively, with over 90% of the particles being within the measured size thus confirming the narrow size distribution. CPMV (uncoated particles) have an average diameter of 28 nm and a surface charge of ca. 13 mV [38].

### Surface functionalization and UV–visible studies

**Figure 3:**
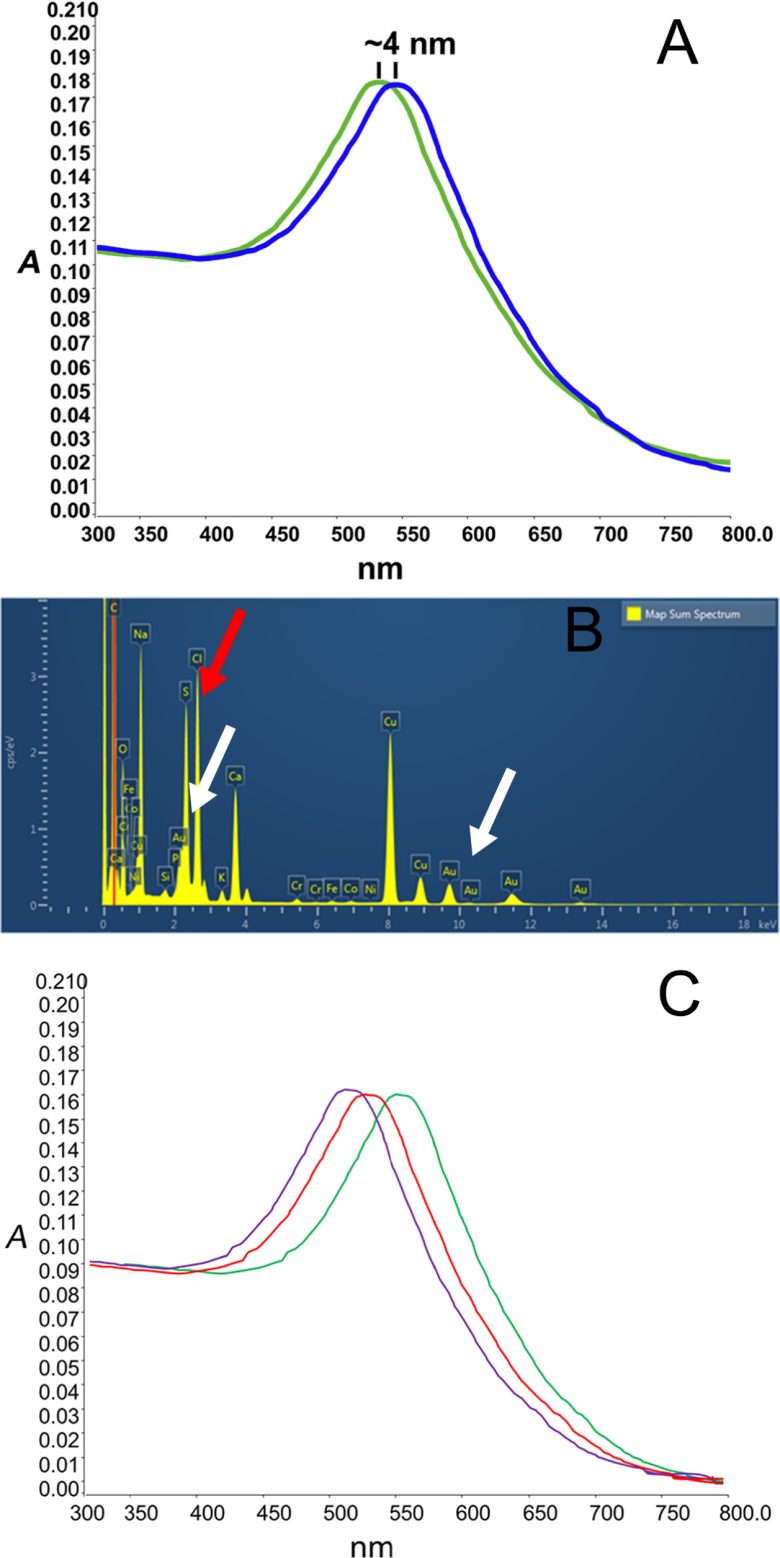
(A) UV–vis spectrum of 50 nm unconjugated Au-CPMV (green) and antibody-labeled Au-CPMV particles (blue). The spectrum shows a red-shift of 4 nm, while preserving the overall shape and intensity of the spectrum, confirming successful coupling of the antibody to the Au-CPMV surface. (B) EDX analysis confirms the presence of gold (white arrows) and sulfur (red arrow), Si and Cr signals are from the sample holder. (C) UV–vis spectrum of the SPR bands of Au-CPMV; λ_max_ = 535 nm for particles with a diameter of 50 nm (purple line), λ_max_ = 552 nm for particles with a diameter of 70 nm (red line) and λ_max_ = 572 nm for particles with a diameter of 100 nm (green line).

One of the objectives of the present work was the development of a smart cell-specific contrast agent based on the surface modification of Au-CPMV with specific antibodies to target desired cells. Quantification of the amount of antibody attached to the ^VCAM1-PEG5000^Au-CPMV was determined spectrophotometrically at λ = 565 nm using BCA reagent and found to be 1.95 ± 0.18 mg/mL gold. Further, the hydrodynamic radius of the ^VCAM1-PEG5000^Au-CPMV increases from 50.2 ± 3.2 nm to 56.1 ± 2.4 nm after rigorous purification of the antibody-labeled particles suggesting successful modification of their exterior surface. This observed increase in the hydrodynamic radius is consistent with the previous report of particles coated with proteins [39]. In addition, the UV–vis spectrum was used to evaluate the surface functionalization of Au-CPMV. The localized surface plasmon resonance (LSPR) spectrum shifted by almost 4 nm (Figure 3A). This shift of the extinction maximum from 534 nm to 538 nm is a result of an increase in the local refractive index at the Au-CPMV surface as reported in the literature following surface modification with proteins [40] and indicates that the surface of the Au-CPMV particles is “smooth”. The shift would be greater if the surface had an uneven shape. In addition, the 4 nm red-shift of the LSPR peak suggests that the modification of the Au-CPMV surface with antibodies has been successful. This increase in the absorbance shift is expected as the SPR of the AuNPs is sensitive to their interparticle distance and surface state [41]. Furthermore, the LSPR band of Au-CPMV is dependent on the size of the particles. The peak absorbance wavelength increases with the increase of the particle diameter as shown in (Figure 3C). The UV–vis spectrum was used to calculate the concentration of Au-CPMV particles using the Beer–Lambert law with λ = 535 nm and an extinction coefficient ε = 1.8 × 10^10^ M^−1^·cm^−1^ for a particle diameter of 50 nm, with λ = 552 nm and ε = 6.70 × 10^10^ M^−1^·cm^−1^ for a particle diameter of 70 nm, and with λ = 572 nm using ε = 1.57 × 10^11^ M^−1^·cm^−1^ for particles with a diameter of ca. 100 nm. In addition, the EDX spectrum of ^VCAM1-PEG5000^Au-CPMV clearly confirms the presence of gold with a signal at 2.120 keV and 9.712 keV as indicated by the white arrows (Figure 3B) and a strong signal from sulfur (red arrow in Figure 3B) from the linker on the surface of the Au-CPMV particles.

To confirm successful modification of the Au-CPMV with antibodies zeta potential measurements were carried out [39]. The zeta potential of the 50 nm Au-CPMV particles decreased from −45.9 ± 3.1 mV to −53.8 ± 2.4 mV upon antibody coating of the particles. The zeta potential value of ^VCAM1-PEG5000^Au-CPMV becomes more negative [38], which is consistent with the literature [42].

### In vitro fluorescent cell imaging

**Figure 4:**
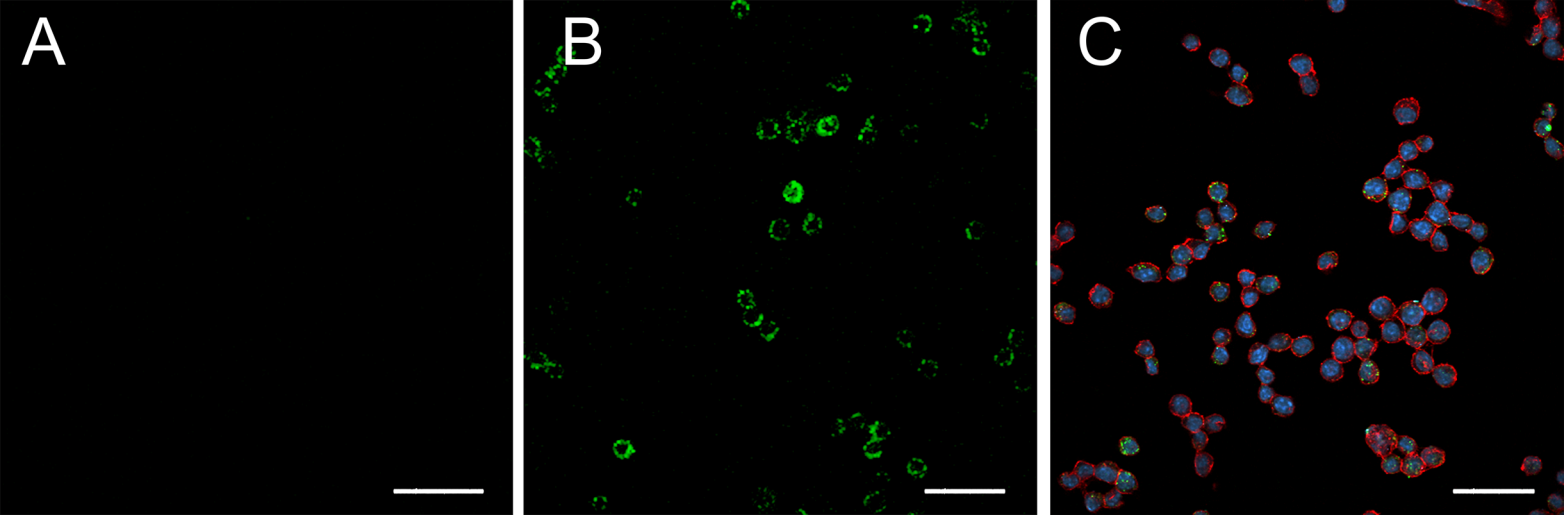
Confocal fluorescence microscopy images of fluorescent labeled ^VCAM1-PEG5000Au^CPMV particles. RAW246.7 actin filaments were labeled with DY-554 phalloidin (red) and DAPI (blue). (A) ^IgG-PEG5000^Au-CPMV (B) ^VCAM1-PEG5000^Au-CPMV showing the binding of the particles to the cells surface and (C) merged image of the cells with ^VCAM1-PEG5000^Au-CPMV showing stained cells and the binding of the particles. Scale bars: 100 μm.

Confocal fluorescence microscopy was performed on the cell lines to demonstrate the specificity and the distribution of the labeled NPs. As shown in Figure 4B, green-fluorescent labeled ^VCAM1-PEG5000^Au-CPMV particles incubated with RAW264.7 cells showed significant and specific binding of the fluorescent labeled antibody on the exterior of Au-CPMV to the surface of the RAW264.7 cells. Fluorescence microscopy confirmed that the ^VCAM1-PEG5000^Au-CPMV can selectively bind to their target, whereas, the ^IgG-PEG5000^Au-CPMV control did not show any fluorescence signal, which is indicative that no binding to the surface of the cells occurred (Figure 4A). The merged confocal microscopy image in Figure 4C demonstrates the successful attachment of the ^VCAM1-PEG5000^Au-CPMV to the surface of the RAW264.7 cells: ^VCAM1-PEG5000^Au-CPMV in green, blue and red indicate the cell nucleus and the plasma membrane, respectively, the images represent the overall morphology of the RAW264.7 cells. Thus, we conclude that the prepared ^VCAM1-PEG5000^Au-CPMV is an acceptable targeting model for further in vivo studies.

### STEM–EDX elemental maps

**Figure 5:**
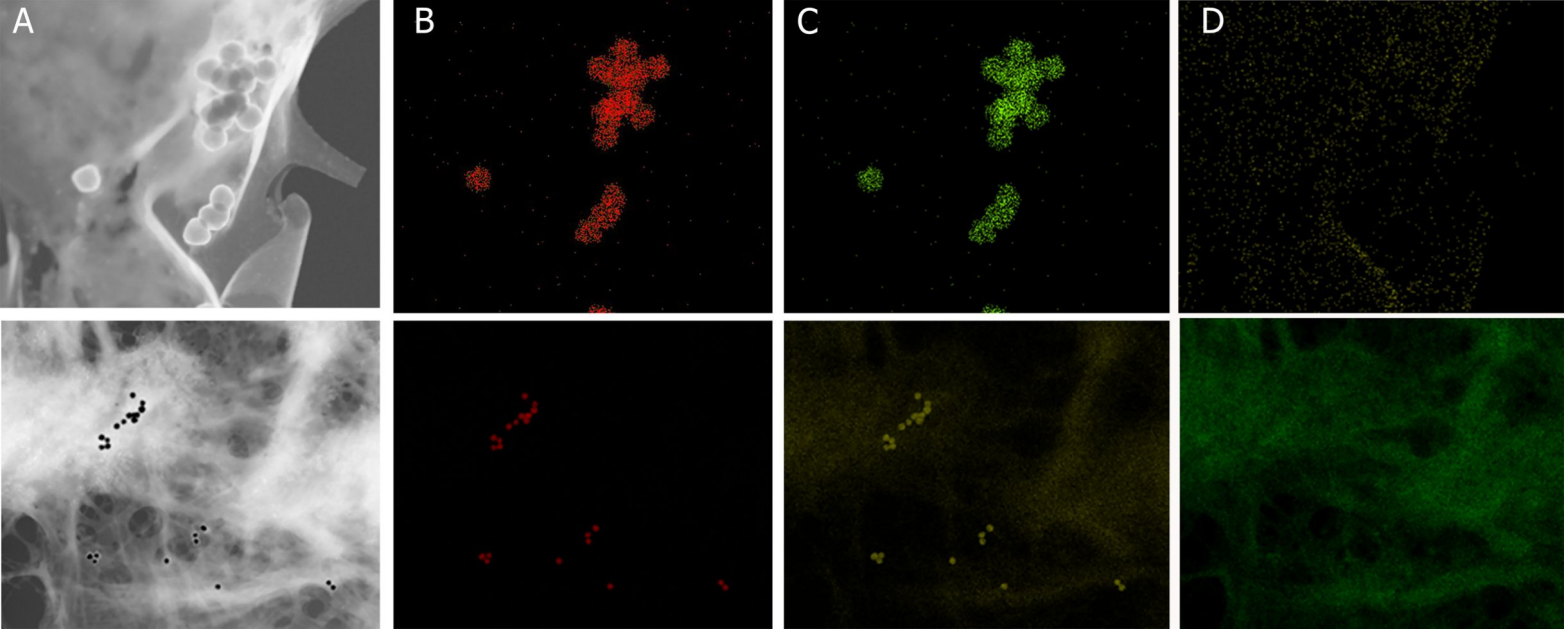
(A) High-resolution TEM image of ^Antibody-PEG5000Au^CPMV; (B) elemental EDX map of gold; the signal is distributed almost uniformly on the same position as the electron-dense particles in (A); (C) sulfur from the linker attachment to the nanoparticles distributed uniformly on the same position as the electron-dense particles in (A); (D) carbon from the biological matrix of the cells. The electron dose is 20*e*^−^/Å^2^ per frame (200 kV, probe current of 691 pA). Top and lower panels represent different magnifications of two independent experiments.

Low-voltage STEM offers a contrast enhancement over conventional TEM analysis due to lower energy (20–30 kV). The higher electron scattering provides better insight into the morphology of materials with low atomic numbers [43]. In the case of imaging Au-CPMV particles in RAW246.7 cells in vitro, the electron-dense particles appeared to be outside the cell and on the cellular surface as shown in (Figure 5A). The images revealed that Au-CPMV particles maintain their original shape and size, indicating that they are resistant to solubilization or oxidation. The dual STEM and EDX spectra from the ^Antibody-PEG5000^Au-CPMV gave useful information about the spatial distribution of gold and sulfur across the cellular surface. The simultaneously acquired EDX spectrum images confirmed that gold and sulfur are at the surface as a consequence of the modification of Au-CPMV with the antibody linker. Furthermore, STEM-EDX analysis provided compositional and topographical 3-D elemental distributions (Figure 5).

### CT Imaging

**Figure 6:**
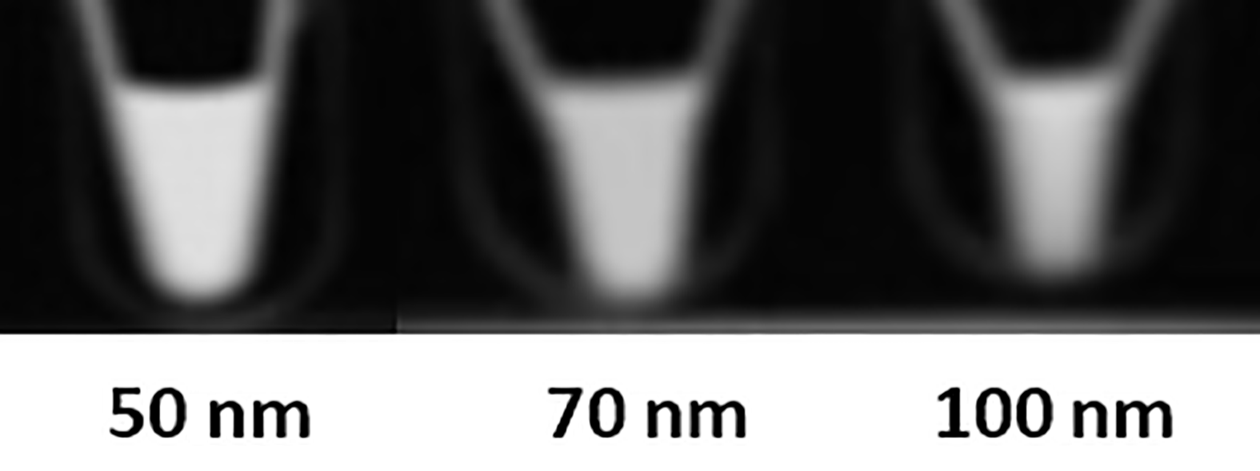
CT images of Au-CPMV particles of different sizes suspended in DD water at, the gold concentration is 200 μg of gold suspended in 200 μl aqueous solution for each sample. The average CT values from three scans for each sample were 183 HU, 133 HU, and 115 HU for the 50 nm, 70 nm, and 100 nm, respectively.

Iodine-containing compounds are routinely used as CT imaging agents [44] due to the high X-ray attenuation of iodine [45]. It has been shown that gold provides on average a three times higher X-ray attenuation per unit weight than iodine [46]. This was the rationale to use AuNPs as CT imaging agents. The Au-CPMV particle sizes of 50, 70 and 100 nm were selected to ensure that the particles will bind specifically to their target tissue without being so small as to induce cell toxicity. This occurs at sizes smaller than 50 nm because of diffusion into the cells. We believe that the Au-CPMV particles have suitable sizes for optimal imaging quality and biocompatibility in clinical applications. Herein, we report the size effect of the three Au-CPMV samples on X-ray attenuation as measured by CT. The generated signals are shown in Figure 6. The average CT number from three scans for each sample was 183 HU, 133 HU, and 115 HU for the 50 nm, 70 nm, and 100 nm particles, respectively. This can be attributed to the increase of the total surface area of all NPs with decreasing particle size. In addition, gold has a significantly higher X-ray attenuation coefficient (at 100 keV: gold 5.16 cm^2^/g; iodine 1.94 cm^2^/g; water 0.171 cm^2^/g) [47–48].

## Conclusion

CT has become an extremely useful imaging tool. CT yields non-invasive, three-dimensional high-resolution images. CT contrast agents have evolved from iodinated molecules to complex nanoparticles. Au-CPMV particles are easy to synthesize while having control over the NPs size. In addition, the ability to tune the surface functionalities allows for the use of such particles in biomedical applications. The Au-CPMV particles reported here exhibit excellent stability over at least almost a year. No visible aggregation nor changes in particles size was observed in samples stored at 4 °C. Their surface can be modified with molecules to enhance tissue targeting. Coupling of fluorescent labeled antibodies to the nanoparticles have enabled cell line studies. As a CT agent, Au-CPMV particles generated on average a signal of 150 HU in a size-dependent manner showing contrast enhancement similar to, or better than, other contrast agents. Au-CPMV-labeled cell tracking has great potential for use in clinical studies.

## Experimental

### Materials

Poly(allylamine hydrochloride) (PAH, *M*_W_ ≈ 15,000), hydrogen tetrachloroaurate trihydrate 99.9%+, sodium chloride, 50 and 100 kDa cut-off Millipore filter membranes, potassium carbonate, sucrose, dimethyl sulfoxide (DMSO; 25%), 2-(*N*-morpholino)ethanesulfonic acid (MES) buffer, isopropanol, tetrachloroauric acid, trypsin, ethylenediaminetetraacetic acid (EDTA), HEPES sodium salt, Triton™ X-100, phosphate-buffered saline (PBS) tablets, and bovine serum albumin (BSA) were purchased from Sigma-Aldrich; potassium carbonate was purchased from BDH; hydroxylamine hydrochloride (99%) was purchased from Lancaster Synthesis; carborundum (fine-grade silicon carbide) was purchased from Parchem; carboxyl-PEG 5000-SH, (1-ethyl-3-(3-dimethylaminopropyl)carbodiimide hydrochloride) (EDC), *N*-hydroxysulfosuccinimide (sulfo-NHS), bicinchoninic acid (BCA) protein assay kit, RPMI, foetal calf serum, and T125 mm tissue culture flasks were purchased from ThermoFisher Scientific; EGM-2 medium was purchased from Lonza. Cell culture medium phenol red-free (high-glucose Dulbecco modified eagle medium (DMEM) supplemented with 10% (v/v) fetal bovine serum (FBS); 100 units/M1 penicillin, gentamicin antibiotic (50 μg/mL), fungizone (1.3 μg/mL) and 2 mM L-glutamine), anti-VCAM1 (FITC-labeled, EPR17010-83; Abcam) and macrophage cell line (RAW264.7) were purchased from American Type Culture Collection (ATCC; Manassas, VA).

### Methods

#### CPMV propagation

CPMV propagation followed our previously published method [38]. Black-eyed peas plant (*Vigna unguiculata*) were grown from seeds, obtained locally, in a greenhouse for 10 days. Primary leaves were rubbed with carborundum (fine-grade silicon carbide abrasive) and treated with CPMV (50 μL of 0.1 mg/mL) suspended in 10 mM sodium phosphate buffer pH 7.0. The leaves were sprinkled with tap water to remove the excess carborundum. 14 days after infection, the infected leaves were harvested and stored at −20 °C prior to use.

#### CPMV isolation

Infected leaves were homogenized to extract CPMV particles following our previously published protocol [34].

#### CPMV-poly(allylamine) hydrochloride (^PAH^CPMV)

^PAH^CPMV particles were prepared as previously reported [30]. CPMV (1 mg/mL) was suspended in double-distilled (DD) water and added dropwise to a freshly prepared aqueous solution of PAH (1 mg/mL; supplemented with 250 mM NaCl) over 5–10 min under continuous stirring at 500–800 rpm at ambient temperature. ^PAH^CPMV particles were washed four times with DD water (15 mL each) on 50 kDa cut-off Millipore filter membranes, followed by dialysis with 12,400 molecular weight cut-off (MWCO) against 10 mM sodium phosphate buffer pH 7.0 for 15 h.

#### Gold hydroxide solution

Gold hydroxide solution was prepared following the published protocol [30]. Hydrogen tetrachloroaurate (HAuCl_4_·3H_2_O) (17.4 mL of 25 mM aqueous solution) was diluted with DD water (982.6 mL), and potassium carbonate (249 mg, 1.8 mM) was added. The solution was aged for 1–2 days in dark (foil wrapped) at 4 °C, during which it changed color from yellow to colorless indicative of gold hydroxide formation

#### Gold-coated CPMV (Au-CPMV)

Particles were prepared following the published protocol [30]. Freshly prepared ^PAH^CPMV (approximately 1 mg/mL) was incubated with gold hydroxide solution (0.8 mL to generate 50 nm particles, 1.1 mL to generate 70 nm particles and 1.5 mL to generate 90 nm particles). The solution was stirred continuously at 500 rpm for 2 h at ambient temperature. Freshly prepared aqueous solution of hydroxylamine hydrochloride was added to a final concentration of 20 mM. The reaction was left to proceed for further 30 min at ambient temperature. The Au-CPMV particles were centrifuged at 5000*g* for 20 min to remove any large aggregates. The supernatant was layered on sucrose gradients (15 mL, 10–70 % (w/v) dissolved in 10 mM sodium phosphate buffer pH 7.0). Sucrose fractions containing Au-CPMV (light blue color) were collected and dialyzed against 10 mM sodium phosphate buffer pH 7.0 using 50 kDa MWCO.

#### ^Carboxyl-PEG5000^Au-CPMV

Freshly prepared Au-CPMV (1 mg/mL) suspended in PBS buffer (20 mM sodium phosphate, 150 mM NaCl; pH 7.4) was added to a solution of carboxyl-PEG 5000-SH (spacer arm length 15.8 Å, 10 mg) dissolved in DMSO (1 mL). The reaction was stirred for ca. 12 h at ambient temperature. ^Carboxyl-PEG5000^Au-CPMV particles were dialyzed for 24 h against 100 mM sodium phosphate, 0.15 M NaCl, buffer pH 7.4 using 100 kDa dialysis membranes.

#### ^VCAM1-PEG5000^Au-CPMV

^Carboxyl-PEG5000^Au-CPMV were buffer-exchanged using 14000 kDa dialysis bags in 2-(*N*-morpholino)ethanesulfonic acid (MES) buffer, pH 6.0 for 12–14 h. To ^Carboxyl-PEG5000^Au-CPMV (100 μL, ca. 1 mg/mL), aqueous EDC (50 μL, 200 mM) and aqueous (*N*-hydroxysulfosuccinimide)sulfo-NHS (200 μL, 800 mM) was added. The reaction was left to proceed for 1 h at ambient temperature (25 °C) then precipitated with isopropanol (500 μL). Activated ^Esterfied-PEG5000^Au-CPMV was resuspended in anti-VCAM1 (FITC-labeled, 20 μL of 1 mg/mL) antibody solution in PBS, pH 7.4 (EPR17010-83; Abcam) and reacted at 4 °C overnight (ca. 15 h). ^VCAM1-PEG5000^Au-CPMV was centrifuged at 5000*g* and washed four times with DD water to remove unbound antibodies. Particles were purified on PD-10 columns pre-equilibrated with 10 mM sodium phosphate buffer (pH 7.0). ^IgG-PEG5000^Au-CPMV were prepared as a negative control following the same procedure.

#### Antibody quantification

Bicinchoninic acid (BCA) protein assay kit from ThermoFisher Scientific was used according to the manufacturer’s instructions [49]. ^VCAM1-PEG5000^Au-CPMV and ^IgG-PEG5000^Au-CPMV (200 μL of 0.1 mg Au) and BCA reagent (200 μL) were mixed together and incubated at 60 °C for 10 min. The samples were left to cool for 30 min and then centrifuged at 14000*g* for 40 min (Thermo Scientific CL10 Centrifuge) to pellet the particles. The supernatant BCA dye absorbance was measured at λ = 565 nm using a microplate reader. The change in absorbance is a consequence of the reduction of Cu^2+^ to Cu^+^ and, thus, an indicator of the presence of protein.

#### Murine macrophage (RAW264.7)

**Cell culture:** A mouse monocyte/macrophage cell line (RAW264.7), was purchased from American Type Culture Collection (ATCC; Manassas, VA). RAW264.7 cells were plated in T125 mm tissue culture flasks at 6000 cells/cm^2^ in growth medium phenol red-free following the published protocol [50]. All cells were cultured in a humidified incubator at 95% humidity and 5% CO_2_ maintained at 37 °C. For experiments cells were seeded the day prior to the incubation with the NPs at 3.5 × 10^4^ cells/cm^3^ of growth surface and were used between passages 2 and 3. Subculture occurred after 60–70% confluence after trypsinization (0.025% trypsin, 0.5 mM EDTA, 10 mM HEPES buffer pH 6.5).

**RAW264.7 cell labeling and confocal microscopy:** Cells of a murine endothelial line (100 μL of 1 × 10^6^ cells/mL, RAW264.7) were cultured on a glass coverslip, kept in a six-well plate for 10–12 h prior to the NP addition. ^VCAM1-PEG5000^Au-CPMV (100 μg/mL) was incubated with the cells on the coverslip for 2 h at 4 °C. Coverslips were washed three times with 10 mM sodium phosphate buffer pH 7.0 to remove free NPs. Cells were fixed in 4% paraformaldehyde/PBS (pH 7.0) for 15 min at ambient temperature (25 °C). Cells were rinsed three times for 5 min with PBS (10 mL) and incubated in 0.2% Triton X-100 for further 10 min. After three five-minute rinses with PBS and preincubation with 2% BSA to block nonspecific staining, cells were stained with fluorescein phalloidin (red) (5 to 10 μg/mL) for 20 min to stain F-actin. After three additional five-minute washes with PBS (10 mL), the nuclei were stained with 4′,6-diamidino-2-phenylindole (DAPI) (1 μg/mL in PBS) for 15 min. Samples were washed three times with 10 mL of PBS and analyzed with a fluorescence microscope (Cytation Cell Imager; BioTek Instruments, Inc).

#### Transmission electron microscopy

TEM images were recorded using a Titan FEI microscope, operating at 300 kV and fitted with a post-column Gatan Tridiem GIF 863 imaging filter. Samples were dispersed in water at a concentration of 0.01–0.05 mg/mL and deposited on 400 mesh carbon grids (SPI supplies), samples were air dried prior to imaging.

#### Energy-dispersive X-Ray spectroscopy

A FIB Scios system was used combined with a scanning electron microscope (SEM) and a focused ion beam equipped with X-MaxN 50 mm^2^ EDS system to measure 0.3–3 μm with a detection limit of ca. 1%. The sample was placed at a 52° tilt angle and at a eucentric height (or WD) of 7–10 mm from the pole piece. The Auger electrons were set to ca. 1 nm, secondary electrons to 100 nm and inelastically backscattered electrons to 1 μm under vacuum.

The EDX data was processed by Aztec software from Oxford Instruments. Images were recorded on CCD camera with mapping resolution of 2048 × 1600. The beam was selected with accelerating voltage for imaging, beam current 100 pA at 30 kV and a spot size of 5–6, fast scan rate of dwell time (0.1–0.3 μs), detector ETD (SE) and a working distance (FWD).

#### UV–visible spectroscopy

The absorption measurements were recorded on a PerkinElmer Lambda 25 spectrometer. CPMV concentration was determined using the Beer–Lambert equation with a mass extinction coefficient of 8.1 mL·mg^−1^·cm^−1^ [51]. Au-CPMV concentration was determined from peak SPR wavelength λ = 535 nm using ε = 1.8 × 10^10^ M^−1^·cm^−1^ for a particle diameter of 50 nm, SPR wavelength of λ =552 nm using ε = 6.70 × 10^10^ M^−1^·cm^−1^ for a particle diameter of 70 nm, and from SPR peak wavelength λ = 572 nm using ε = 1.57 × 10^11^ M^−1^·cm^−1^ for particles with a diameter ca. 100 nm [36]. Spectroscopic analyses were recorded at ambient temperature (21–28 °C) using quartz cuvettes with an optical path length of 1 cm.

#### Dynamic light scattering

DLS measurements were carried out on a Wyatt DynaPro^TM^ DP-801 system coupled with Dynamics V7.0.0.95 software. Measurements were recorded with the following settings: 20 mW He–Ne laser, λ_0_ = 780 nm, scattering angle θ = 90°, molar refractive index of 1.33; viscosity of 0.8872 at 25 °C; the attenuator was set up automatically and ranged between 6 to 9. Corresponding quartz cells were flushed with deionized water followed by a 1% (v/v) aqueous Hellmanex solution (strong alkaline cleaning concentrate made of phosphates and surfactants from Helma Analytics) and air dried prior to being filled with sample solution (500 μL). The outer surface of the cells was wiped gently with a sheet of soft lens cleaning tissue. In total, 10 successive DLS measurements were carried out per sample after 2 min waiting time to allow the solutions to be at rest. The hydrodynamic radius (intensity particle size distribution was used for all measurements) was calculated by the instrument from the translational diffusion coefficient using the Stokes–Einstein equation.

#### Zeta potential measurements

The zeta potential of the particles was measured on a Zetasizer™ NanoZS-90 (Malvern Instruments) equipped with a 4 mW, λ_0_ = 632 nm He–Ne laser operating with a detector angle of θ = 173° degree. The cell voltage of the instrument was fixed to 80 V during measurements. The reference beam had an intensity within 2000 and 3500 kcps. Zeta potential values were reported as an average of three measurements from each sample.

#### Nanoparticle tracking analysis

Hydrodynamic diameters of the nanoparticles and their concentration (particle numbers) were measured by nanoparticle tracking analysis (NTA) using a NanoSight LM10 with a laser module LM14 set at a wavelength of 532 nm, NTA 2.3 build 0033 analytical software (Malvern Instruments Ltd, Malvern) and a high-sensitivity sCMOS camera. Particles were suspended in PBS buffer pH 7.4. The samples were injected in the sample chamber with sterile syringes until the solution reached the tip of the nozzle. Ten 30 s videos using a camera level of 7 and a detection threshold of 5, were recorded for each sample and the software was used to estimate concentration and NP size. Measurements were recorded at ambient temperature with camera setting of 380 gain and shutter speed of 15 ms with auto particle detection settings. The instrument was calibrated with 100 nm standard polystyrene beads with known concentrations prior to sample recordings.

#### Scanning transmission electron microscopy

A FEI Titan 80-300 TEM/STEM (spherical aberration corrector *C*_s_ ≈ 1.2 mm) operating at 300 kV and equipped with EDAX detector for X-ray analysis and elemental mapping was used. The microscope objective lens is a FEI Tecnai “Super Twin”. The CCD Gatan Orius SC200D camera located above the viewing chamber is a 4K (2048 × 2048) pixel cooled CCD. The high-angle annular dark field scanning transmission electron microscopy (HAADF-STEM) images further confirmed the existence of dimeric structures (Figure 5A), composed of a brighter core and a darker attachment. STEM-energy dispersive X-ray (EDX) elemental mapping measurements were conducted to analyze chemical composition.

#### Fluorescent imaging of labeled cells

^Antibody-PEG5000^Au-CPMV particles of concentration 50 μg Au/mL were incubated with cells as described above using 20 × 10^3^ cells. Images were recorded on Olympus IX 81 Inverted fluorescence microscope using LUC PLAN 40× objective (numerical aperture 0.6; Olympus). Images were taken using a back-illuminated electron multiplying charge-coupled camera (Andor Technology, Belfast, Northern Ireland)

#### Computed tomography (CT) scanning

Scanning was performed three times (different days) on a multi-slice GE CT (Optima CT660) scanner (GE MEDICAL SYSTEMS) using clinical settings for helical brain scanning (80 kVp and 330 mAs) in a coronal plane to the tubes-containing nanoparticles with in-plane resolution of 0.5 × 0.5 mm and slice thickness of 5 mm. Images were retrospectively reconstructed into an isotropic voxel of 0.5 mm^3^ and loaded into the ImageJ software (https://imagej.nih.gov/ij/ ) in analyze format to calculate the average signals (mean CT number) for each sample from the three scans.
